# Consecutive Isoproterenol and Adenosine Treatment Confers Marked Protection against Reperfusion Injury in Adult but Not in Immature Heart: A Role for Glycogen

**DOI:** 10.3390/ijms19020494

**Published:** 2018-02-07

**Authors:** Martin Lewis, Adrian Szobi, Dirki Balaska, Igor Khaliulin, Adriana Adameova, Elinor Griffiths, Clive H. Orchard, M.-Saadeh Suleiman

**Affiliations:** 1 Bristol Medical School, University of Bristol, Bristol BS8 1TH, UK; I.Khaliulin@bristol.ac.uk (I.K.); m.s.suleiman@bristol.ac.uk (M.-S.S.); 2 Department of Pharmacology and Toxicology, Faculty of Pharmacy, Comenius University in Bratislava, 814 99 Bratislava, Slovakia; adrian.szobi@gmail.com (A.S.); adameova@fpharm.uniba.sk (A.A.); 3 School of Biochemistry, University of Bristol, Bristol BS8 1TH, UK; d.balaska@gmail.com (D.B.); elinor.griffiths@uhbristol.nhs.uk (E.G.); 4 School of Physiology, Pharmacology & Neuroscience, University of Bristol, Bristol BS8 1TH, UK; clive.orchard@bristol.ac.uk

**Keywords:** ischemia, isoproterenol, adenosine, cAMP, immature heart, cardioprotection, glycogen

## Abstract

Consecutive treatment of adult rat heart with isoproterenol and adenosine (Iso/Aden), known to consecutively activate PKA/PKC signaling, is cardioprotective against ischemia and reperfusion (I/R). Whether this is cardioprotective in an immature heart is unknown. Langendorff–perfused hearts from adult and immature (60 and 14 days old) male Wistar rats were exposed to 30 min ischemia and 120 min reperfusion, with or without prior perfusion with 5 nM Iso for 3 min followed by 30 μM Aden for 5 min. Changes in hemodynamics (developed pressure and coronary flow) and cardiac injury (Lactate Dehydrogenase (LDH) release and infarct size) were measured. Additional hearts were used to measure glycogen content. Iso induced a similar inotropic response in both age groups. Treatment with Iso/Aden resulted in a significant reduction in time to the onset of ischemic contracture in both age groups whilst time to peak contracture was significantly shorter only in immature hearts. Upon reperfusion, the intervention reduced cardiac injury and functional impairment in adults with no protection of immature heart. Immature hearts have significantly less glycogen content compared to adult. This work shows that Iso/Aden perfusion confers protection in an adult heart but not in an immature heart. It is likely that metabolic differences including glycogen content contribute to this difference.

## 1. Introduction

Cardiac ischemia and reperfusion (I/R) injury is an important factor that contributes to morbidity and mortality during open heart surgery [1,2]. Therefore, the search continues for potent cardioprotective interventions that can be used to protect the adult and pediatric heart. We relatively recently described cardioprotective intervention termed temperature preconditioning (TP) [3] that confers protection by increasing myocardial cAMP and subsequent activation of Protein Kinase A (PKA) followed by activation of Protein Kinase C (PKC) [4]. This sequence of events was confirmed by our finding that the PKA inhibitor H-89 attenuated PKC activation following TP [4,5] and led us to the formulation of a new cardioprotective pharmacological strategy that involves cAMP/PKA/PKC signaling. Based on this strategy, we have found that the normal adult rat heart can be effectively protected against I/R injury by transient and consecutive treatment with clinically relevant doses of isoproterenol and adenosine (Iso/Aden) [6]. This intervention has also been shown to protect hearts when applied during hypothermic cardioplegic arrest [7] and in rat models of heart failure and aging [6,8]. Interestingly, this treatment results in a decrease in glycogen content in the myocardium [4,6]. Glycogen breakdown and subsequent accumulation of glucose-6-phosphate and low pH during ischemia triggers dissociation of mitochondrial hexokinase 2 which can reduce mitochondrial permeability transition pore (MPTP) opening and cardiomyocyte death [9,10,11]. What is not known is whether this intervention can protect the immature heart. This issue is further complicated by the fact that unlike adult heart, the vulnerability of postnatal hearts to I/R changes during development and follows a triphasic pattern in rats with 1 and 14–21 days old being most resistant to I/R injury [12,13,14,15]. Clinical research has also shown developmentally-related differences in cardiac vulnerability to cardiac I/R in patients undergoing open heart surgery [16,17]. The underlying mechanisms are not known but may include developmental changes in caloric intake/body mass [15,18] and in Ca^2+^ mobilization. This metabolic link could also involve cAMP/PKA signaling pathway, which is important in the regulation of cardiac Ca^2+^ cycling. In fact, cell cycle arrest of cardiomyocytes in G1 phase could involve β-adrenergic receptors (β-AR) [19]. The aim of this work was to investigate whether an immature heart of 14 days postnatal age (selected to have relatively high resistance to I/R) can also be protected by Iso/Aden treatment.

## 2. Results

### 2.1. Effect of Isoproterenol on Cardiac Function in Adult and Immature Heart 

**Figure 1:**
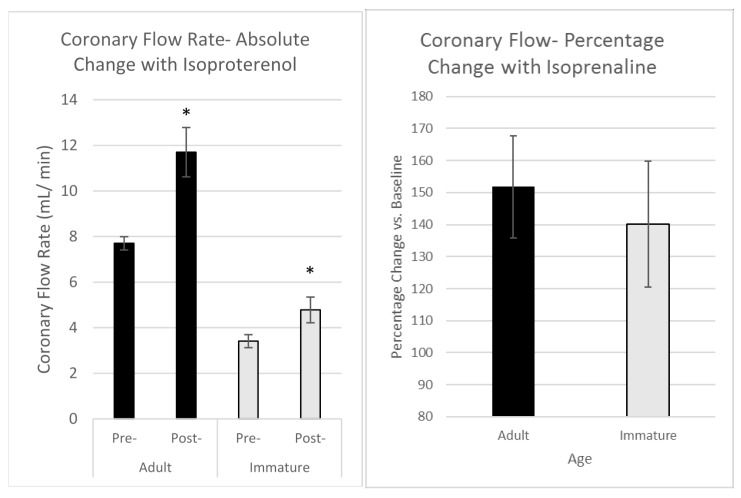
Maximal change in coronary flow measured in adult and immature hearts prior to (pre-) and following (post-) perfusion with 5 nM isoproterenol for 3 min. (**Left**) panel—absolute changes, (**Right**) panel—proportionate changes relative to control for each age group. Data points are mean ± SEM. * *p* < 0.05 vs. corresponding pre-values.

The coronary flow rate (CFR) (Figure 1) was significantly increased in both groups following administration of 5 nM isoproterenol. In the adult group, the CFR increased from an average value of 7.7 ± 0.6 mL/min after equilibration to 11.7 ± 1.11 mL/min; the immature group showed an increase from 3.6 ± 0.26 mL/min to 4.8 ± 0.57 mL/min after Iso perfusion. The percentage increase was similar for both groups and there was no significant (intergroup) difference in the percentage increase (151.9% vs. baseline in the adult and 140.2% in the immature animals). 

**Figure 2:**
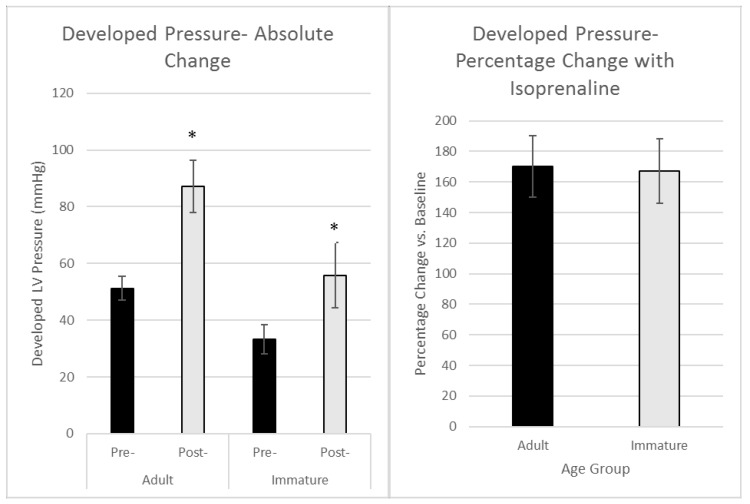
Maximal change in left ventricular developed pressure measured (LVDP) in adult and immature hearts perfused with 5 nM isoproterenol for 3 min. (**Left**) panel; LVDP prior to heart perfusion with isoproterenol (Pre-) and at peak of LVDP following addition of isoproterenol (Post-). (**Right**) panel; proportionate changes of LVDP during heart perfusion with isoproterenol relative to control for each age group. Data points are mean ± SEM of mean. * *p* < 0.05 vs. corresponding pre- values.

There was a significant increase in Left Ventricular Developed Pressure (LVDP, Figure 2) in both the adult (from 50.00 ± 3.88 to 87.22 ± 9.2 mmHg) and immature hearts (from 32.44 ± 3.99 to 55.88 ± 11.5 mmHg). However, there was no difference between the groups in the percentage increase in LVDP (174% vs. 172% for adult and immature, respectively). 

### 2.2. Effects of Isoproterenol/Adenosine Pre-Treatment on Ischemic Contracture

**Figure 3:**
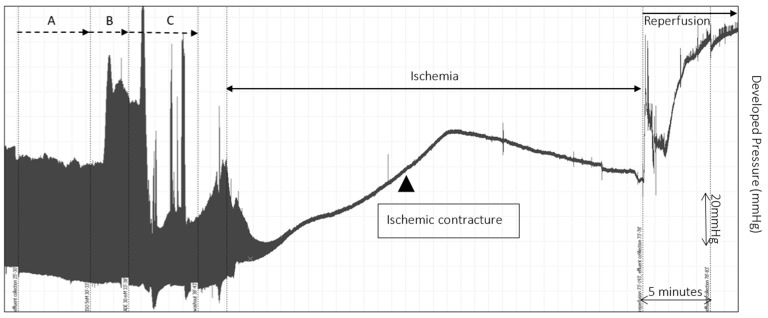
A representative experimental trace of changes in LV pressure (mmHg) measured before interventions (A), during Isoproterenol (B) and Adenosine (C) and during Ischemia and early reperfusion. Arrowhead shows the gradual increase in developed pressure that is a measure of ischemic contracture (rigor). *Y*-axis shows the developed pressure in mmHg and *X*-axis shows time in minutes. Scales are shown on the right-hand side of the trace.

**Figure 4:**
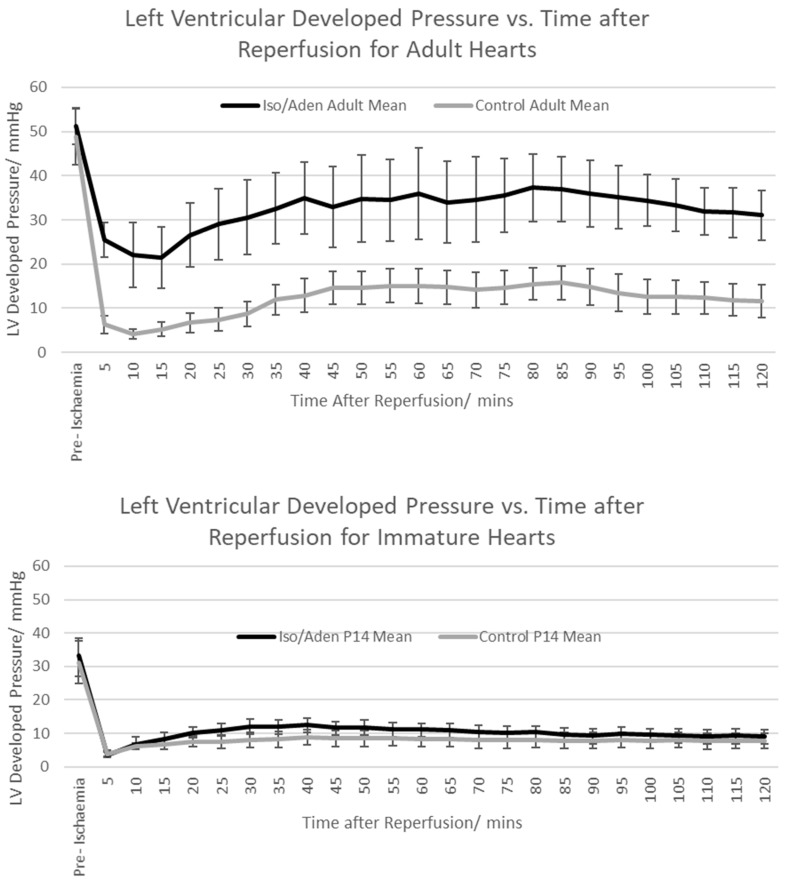
Time course of mean LVDP prior to and following reperfusion measured at 5 min intervals. Plotted as mean pressure ± SEM. (**Top**) panel—adult hearts; (**lower**) panel—14-day old immature hearts.

A representative trace of a single experiment, and mean LVDP during the time following reperfusion, are shown in Figure 3 and Figure 4. Figure 3, the trace from a single perfusion experiment, shows the typical pattern for an ischemic heart; that is, an LVEDP measurement which falls sharply on cessation of coronary flow, but over time during ischemia comes to a peak. This peak is known as ischemic hypercontracture.

Table 1 shows a summary of the changes in end diastolic pressure (LVEDP) during ischemia for both adult and immature heart. In both age groups, the time to onset of ischemic contracture was significantly shorter in hearts pre-treated with Iso/Aden. The peak rise in LVEDP was two-fold lower (Table 1) in the drug-treated adult heart compared to control adult heart and by 40% lower in the treated immature group of hearts. A change was also seen in the time to peak ischemic contracture, with the intervention in the immature group more than halving this duration. Similarly, the increase in LVEDP after 30 min of ischemia (Table 1) was twice as high in adult control vs. adult treated heart; in the immature group, a similar pattern was observed.

### 2.3. Effects of Isoproterenol/Adenosine Pre-Treatment on Outcome Following Reperfusion

#### 2.3.1. Developed Pressure Following Ischemia and Reperfusion (I/R) Injury in Adults & Immature Hearts

**Figure 5:**
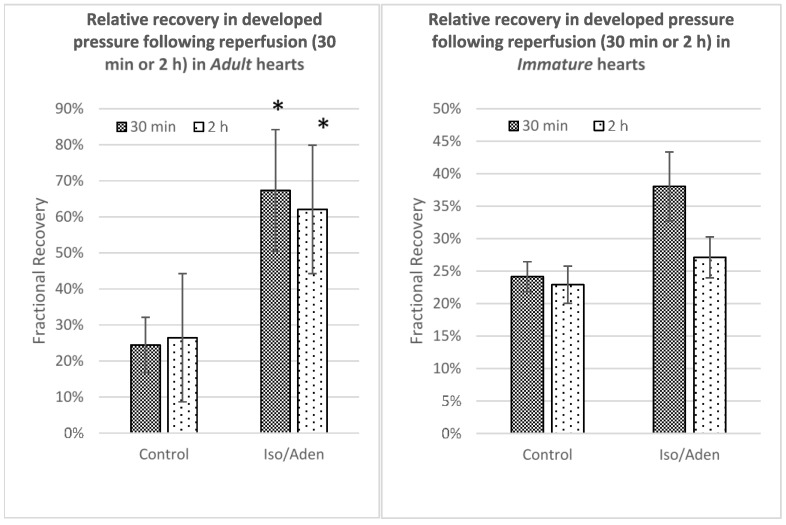
The effect of Iso/Aden on functional recovery after ischemia/reperfusion. Left ventricular developed pressure following ischemia at 30 min and 2 h of reperfusion for adult (**Left** panel) and immature (**Right** panel) hearts comparing control to Iso/Aden intervention. * *p* < 0.05 vs. Control. Data points are mean ± standard error of mean.

Figure 5 shows LVDP in adult and immature hearts exposed to I/R, with and without intervention, measured, at 30 min and 2 h following reperfusion. In adult hearts, LVDP was significantly higher in the Iso/Aden treated group than in the control group at both 30 min (67.3 ± 16.9% vs. 24.4 ± 7.7%) and 2 h (62.1 ± 18.7% vs. 26.5 ± 15.0%). However, in the immature hearts there was no significant difference, i.e., no improvement in functional recovery, in the Iso/Aden group compared to control (at 30 min 38.1 ± 5.3% vs. 24.1 ± 2.3%; at 2 h 27.1 ± 3.2% vs. 22.9 ± 2.9%). 

#### 2.3.2. Lactate Dehydrogenase (LDH) Release Following I/R Injury in Adults & Immature Hearts

**Figure 6:**
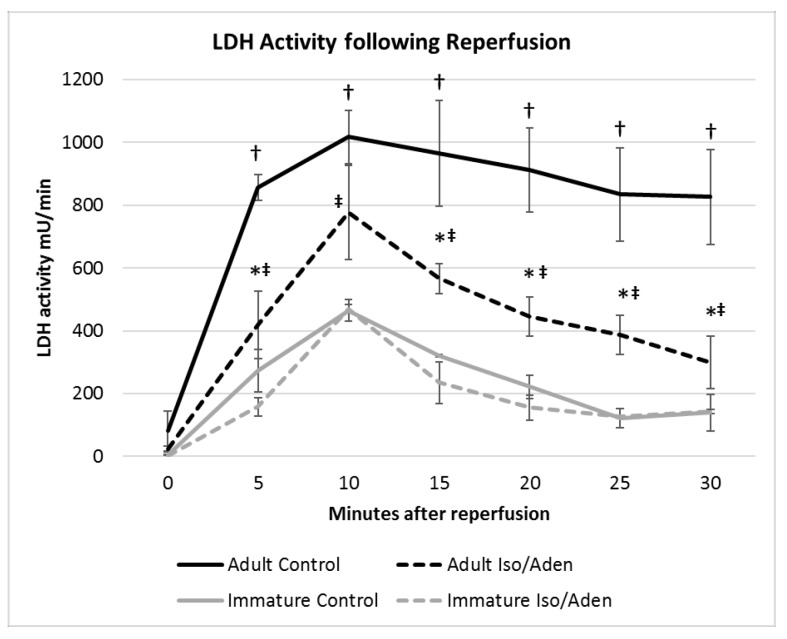
The effect of Iso/Aden on LDH release after ischemia/reperfusion. LDH activity vs. time after reperfusion commenced for intervention (Iso/Aden treatment) and control experiments in the adult and immature groups. Data normalized to the coronary flow rate during the collection of each fraction. There was no difference within the immature group whereas there was a significant decrease in LDH release in treated adult hearts. Time = 0 is from a pre-ischemia fraction. Data shown as Mean ± standard error. * = *p* < 0.05 vs. Adult Control. † = *p* < 0.05 vs. immature control, ‡ = *p* < 0.05 vs. immature hearts treated with isoproterenol/adenosine.

Figure 6 shows that time dependent release of LDH during early reperfusion was significantly greater in adult control hearts compared to immature counterparts, and was reduced by Iso/Aden pre-treatment in adult, but not immature. After normalization for CFR, the area under the curve vs. time for LDH release for both age groups was calculated. The total area was significantly reduced in pre-treated adult hearts compared to control (25,202 vs. 13,783 mU), but was not significantly different in pre-treated and control immature heart (7365 vs. 6080 mU). 

#### 2.3.3. Infarct Size Following I/R Injury in Adults & Immature Hearts

**Figure 7:**
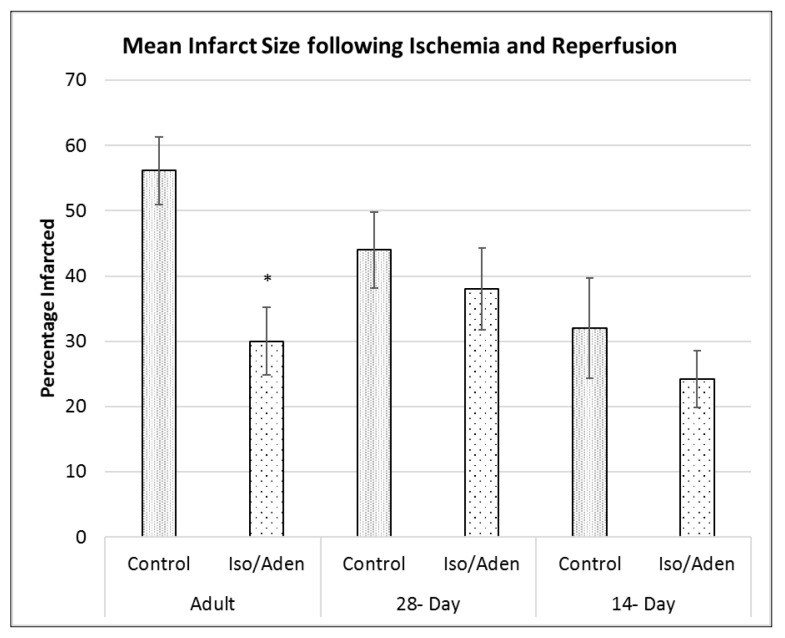
The effect of Iso/Aden on infarct size after 30 min ischemia and 2 h reperfusion. Infarct size in adult, young adult and immature hearts with or without pre-treatment with Iso/Aden. Data presented as mean ± standard error. * *p* < 0.05 vs. Control of the same age group.

Pre-treatment with Iso/Aden decreased the area of infarcted myocardial tissue in adult hearts (Figure 7) from 56 ± 5% to 30 ± 5%. Interestingly, the infarct size was smaller in the control immature hearts than in the control adult hearts, reflecting the same pattern of vulnerability as with LDH release. Following pre-treatment there was a small, non-significant, decrease in the immature group (32.0 ± 7.6% vs. 24.2 ± 4.3%). As the infarct size even in the control group for the immature animals was relatively small, an additional intermediate group of 28 day old hearts was also used (5 control, and 5 Iso/Aden). These hearts did not show a significant difference in infarct size following pre-treatment (control 44.00 ± 5.8% vs. interventions 38.5 ± 3.3%). Intra-group comparison shows increasing vulnerability to infarction, and increasing protection by Iso/Aden pre-treatment with increasing age after 14 post-natal days.

### 2.4. The Effect of Age on Cardiac Glycogen Content

**Figure 8:**
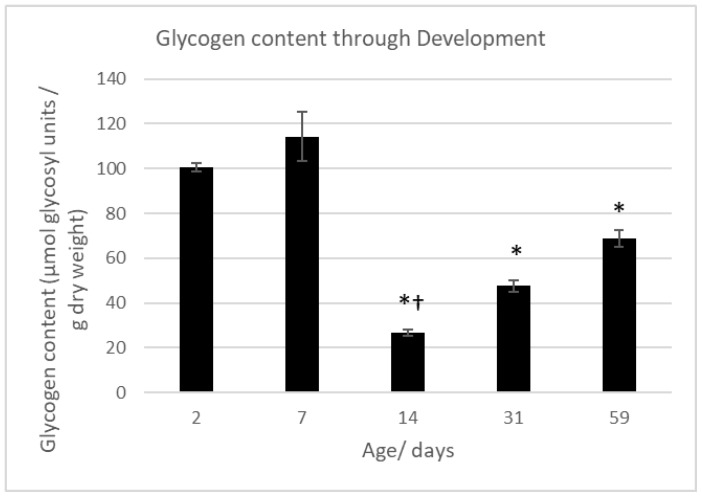
Glycogen levels in hearts during development. Data analysed by 1-way ANOVA with Bonferroni’s post-hoc test. * = *p* < 0.05 vs. both 2 days of age and 7 days of age. † = *p* < 0.001 vs. adult hearts. Data points represent mean ± standard error.

Figure 8 shows glycogen content in 5 age groups: 2 neonatal, immature, young adult and adult. Neonatal hearts had more glycogen than the other age groups. However, there was a marked decrease in this parameter by day 14, to ~26% of the neonatal level, before it subsequently increased to 47% of neonatal levels by 31 days post-natal, and 69% at 59 days. 

## 3. Discussion

### 3.1. Isoproterenol Triggers Similar Inotropic Response in Both Age Groups

This work shows that β-AR stimulation using perfusion with isoproterenol at 5 nM triggers a relatively similar physiological response, of inotropy and increased coronary flow rates, in adult and in immature hearts (Figure 1 and Figure 2). Thus, even though there are marked differences in the response to an ischemic insult between these two groups [12,13,14,15], this is not accounted for in the end physiological outcome from stimulation with Iso. There are, however, conflicting reports showing that the cardiac effects of Iso are different between adult and neonates. This includes desensitization following sustained long periods of activation of β-AR, which interestingly, is not seen in cardiac preparations from the 6 day old rat in contrast to the 15, 25-day old and adult rats [20,21]. 

### 3.2. Time of Onset to Ischemic Contracture Is Reduced Following Pre-Treatment with Isoproterenol/Adenosine

The pattern of change in LVEDP during ischemia was similar for both control groups (Table 1). with the time to onset of ischemic contracture not significantly different in adult and immature control hearts. As expected, the time to the start of ischemic contracture was significantly shorter in both adult and immature groups when pre-treated with Iso/Aden.

It has been suggested that ischemic contracture can be triggered by low levels of Mg-ATP [22]. The observation of a reduced time to onset of contracture with this pharmacological treatment is similar to that reported for ischemic preconditioning [7], which was attributed to loss of glycogen and faster rate of ATP decline during ischemia in these hearts [3,23,24]. Similarly, earlier reports have shown that higher glycogen content is associated with later onset of ischemic contracture [3,23,24]. Our data, showing an earlier onset and decreased glycogen content in immature hearts compared to adult is consistent with this idea which would seem to indicate lower glycogen content being responsible for this observation, and is confirmed by our findings on the glycogen content of hearts of these age groups (Figure 8) and in works of others [25,26]. Below, we discuss the implication of this fact for heart resistance to I/R injury. 

### 3.3. Pre-Treatment with Isoproterenol/Adenosine Protects Adult but Not Immature Heart after I/R

This study confirms our previous observation [4,6] that pre-treatment of adult rat heart with transient consecutive administration of clinically relevant concentrations of Iso and Aden is cardioprotective. However, this intervention did not protect immature hearts exposed to a similar cardiac insult despite both age groups showing similar inotropic responses to Iso perfusion. One major difference between the adult and immature heart is the higher resistance of the immature heart to I/R (Figure 7 and see Introduction). It can be argued therefore that since the same ischemic duration was associated with relatively lower reperfusion injury in the immature heart, which may have masked any protection. However, even at 28 days (Figure 7), when the control group has a larger infarct size than immature, Iso/Aden still did not confer significant protection against I/R.

The observed findings of this study can be explained, at least in part, by consecutive activation of PKA and PKC (protein kinases known to be affected by Iso and Aden) and their respective downstream proteins. Indeed, cyclic AMP-induced PKA activation has been implicated in the cardioprotective effect of ischemic preconditioning [27,28]. In our experiments, glycogen depletion could partially explain the cardioprotective effect in Iso/Aden-treated adult hearts. During ischemia, anaerobic glycolysis and glycogenolysis are activated leading to acidosis and accumulation of glucose-6-phosphate (G6P) in myocardium. Hence, the reduced level of glycogen would lead to less acidosis and reduced G6P accumulation, as it happens during ischemic preconditioning [11], stimulating the binding of hexokinase II (HKII) to mitochondria and inhibiting the MPTP (mitochondrial permeability transition pore). In contrast, in control hearts, elevated level of G6P and low pH during ischaemia would lead to dissociation of HKII from mitochondria [11] and consequently, opening of the MPTP. This causes irreversible reperfusion damage to myocardium ([29] and see Introduction). 

### 3.4. Low Glycogen Content in Immature Heart Could Blunt Cardioprotection with Isoproterenol/Adenosine

Unlike adult hearts, the immature heart has a lower level of glycogen content which is depleted relatively quickly during index ischemia as seen by onset of ischemic contracture. More importantly is the finding that peak ischemic contracture was reached in a very short period of time compared to control or adult hearts. Thus, it is likely that Iso/Aden pre-treatment, by depleting the relatively lower levels of glycogen at an early stage during index ischemia, blunted any expected potential benefits from this intervention, probably due to a significant dysfunction of ATP-dependent ion channels [30]. Although the role of HKII association and dissociation to mitochondria and the link to glycogen content has been addressed, little is known about this in immature hearts. 

## 4. Materials and Methods

### 4.1. Animals & Ethical Approval

Male Wistar rats were used for all experiments. For experiments involving adult rats (control *n* = 8, Iso/Aden *n* = 7), animals of approximately 60 days of age were used; these were obtained from Charles River Laboratories (Margate, Kent, UK). Rat pups aged 14 days of age were obtained from the University of Bristol Animal Services Unit and used for the “immature” experiments (control *n* = 5, Iso/Aden *n* = 6). Additional age groups were used for assays of glycogen content and included 2 days (*n* = 3 samples each with 3 pooled hearts per sample), 7 days (*n* = 3 samples each with 3 pooled hearts per sample), and *n* = 6 for14 days, 31 days and for adult. A young adult group aged 28 days (*n* = 6 for both a control and an Iso/Aden intervention arm) was also included for assays of cardiac vulnerability post-hoc. Rats were stunned by concussion and killed by cervical dislocation, and the heart was rapidly removed. 

All housing and experiments were approved by the University of Bristol Animal Welfare Ethical Review Board (ethical approval number (UIN) UB/15/017 most recently approved 14 April 2016), and conducted in a manner compliant with the Animals (Scientific Procedures) Act 1986 of the United Kingdom and consistent with the Guide for the Care and Use of Laboratory Animals (1996, published by National Academy Press, 2101 Constitution Ave. NW, Washington, DC, USA).

### 4.2. Heart Perfusion

Once the heart was excised, the aorta was cannulated using a 16G diameter cannula for adult rats, and 24G for the immature group. Hearts were then retrogradely perfused at 37 °C with Krebs–Henseleit (KH) buffer (composed of 120 mM NaCl, 25 mM NaHCO_3_, 11 mM glucose, 1.2 mM KH_2_PO_4_, 1.2 mM MgSO_4_, 1.2 mM CaCl_2_, and 4.8 mM KCl) equilibrated with 95% oxygen/5% carbon dioxide using a Langendorff system (AD Instruments, Sydney, Australia). A Powerlab system with Chart 5 software for Windows (AD Instruments, v5.5.6), was used to provide negative feedback control of the perfusion rate.

Two pressure transducers were connected to the perfused heart to measure the hemodynamic parameters. One measured the coronary perfusion pressure, which was held at 80 mmHg for adults, and 65 mmHg for immature, by alteration of coronary flow rate. Left ventricular pressure (LVP) was also monitored, via a pressure transducer connected to a thin plastic balloon inserted into the left ventricle. Left ventricular developed pressure (LVDP) was calculated as the difference between left ventricular systolic pressure (LVSP) and left ventricular end diastolic pressure (LVEDP). 

### 4.3. Ischemia/Reperfusion Injury Protocol

Hearts were allowed to equilibrate for 30 min. In the intervention group—the Iso/Aden group—hearts were perfused with KH containing 5 nM Iso for 3 min followed by 5 min perfusion with KH containing 30 μM Aden, and then a five-minute washout period. Both groups then were subjected to 30 min global ischemia followed by 120 min reperfusion. Functional endpoints and cardiac injury were determined in both control and experimental groups. Hemodynamic activity, such as LVDP and coronary flow (CF), were monitored throughout the experiment. The pre-ischemic data were analyzed for the effect of 5 nM Iso perfusion on physiological function, whilst the peri- and post- ischemic data were also assessed for developed pressure as a measure of cardiac function. Effluent was collected prior to ischaemia and at five-minute intervals during the first 30 min of reperfusion and used to measure lactate dehydrogenase activity. At the end of reperfusion, hearts were stained using triphenyltetrozolium chloride (TTC) to determine the size of infarction as described previously [31]; in which the stained hearts were sliced into six sections, and each slice scanned to produce a digital image. These images were then analysed using the AlphaEase (Alpha Innotec, Kasendorf, Germany) software package to calculate the areas of infarcted and non-infarcted tissue. The ratio of infarcted to total area of the slice was then calculated.

### 4.4. Glycogen Assays

Glycogen levels were measured by a modified method described by Bergmeyer [32]. In addition to adult and immature rats, other age groups were included to investigate neonates (2–7 days), immature (8–22 days) and young adult hearts (4 weeks) [33].

Hearts were excised, rinsed in ice cold phosphate buffered saline (PBS) and blotted on filter paper before freeze clamping using tongs pre-cooled in liquid nitrogen. The frozen hearts were ground to a fine powder and placed in liquid nitrogen. 100 mg of tissue was mixed with 0.2 mL of 30% KOH in a microfuge tube until it dissolved before placing on a heating block at 105 °C for 1 h. The sample was then cooled to 0 °C on ice prior to addition of 0.1 mL of 2% Na_2_SO_4_ followed by 100% ethanol (to give a final concentration of 75%) and left at 4 °C for 24 h. The resuspension was centrifuged on a benchtop Eppendorf Centrifuge 5415D at 16,000× *g* for 2 min, the supernatant discarded and the pellet resuspended in 1 mL of 80% ethanol. The tube was spun again as before and the supernatant was discarded. The pellet was allowed to dry out on a heating block at 40 °C for 24 h. 0.25 mL of 1 M sodium acetate/1 M acetic acid buffer was added with 0.1 mL of 500 μg/mL amyloglucosidase (11.5 units/mL, made in the sodium acetate/acetic acid buffer) followed by 0.75 mL H_2_O. The pellet was resuspended and the tubes incubated at 37 °C for 1 h. 0.2 mL H_2_O was then added and the sample spun as before. The supernatant was assayed spectrophotometrically at 450 nm using a glucose assay kit (Sigma Diagnostic Kit No. 510/510-A, Sigma Aldrich, Darmstadt, Germany). Results were expressed as μmol glycosyl units/g dry weight. For neonatal and immature rats, 3–10 rats were used according to the eventual tissue required, and the hearts pooled to get a good yield. 

### 4.5. Statistics

Following export of the data from the packages initially used to capture results, the data were first processed in Microsoft Excel (Seattle, WA, USA) and subsequent analysis performed with SPSS Statistics (Version 23, IBM Corporation, New York, NY, USA). Functional analysis was performed using a paired two-tailed Student’s *t*-test. Other analysis was performed using unpaired two-tailed Student’s *t*-test for hypothesis testing. In all cases, differences were considered significant where *p* < 0.05. Data analysis for glycogen assays was performed by 1-way ANOVA, and post-hoc analysis performed by Bonferroni’s correction.

## 5. Conclusions

This work shows that unlike the adult heart, the immature heart is not protected by pre-treatment with consecutive Iso/Aden. Relatively lower glycogen content in the immature heart may be responsible for this effect. However, to establish a definitive causal relationship, further study is needed. Such a study would examine changes in glycogen levels caused by isoproterenol and adenosine pre-treatment, as well as the influence of glycogen content on reperfusion injury in hearts from other postnatal age groups.

## Figures and Tables

**Table 1:** Time to key milestones during experimental protocol. Data presented as mean ± SEM.

Age Group	Intervention	Time to Onset of Ischemic Contracture (Seconds)	Time to Peak Ischemic Contracture (Seconds)	Maximal Increase in LVEDP during Ischemia (mmHg)	Increase in LVEDP at the End of Ischemia (mmHg)	Maximal Increase in LVEDP during Reperfusion (mmHg)
Adult	Control	875 ± 32	1125 ± 18	59.41 ± 6.28	41.33 ± 4.66	66.58 ± 8.42
	Iso/Aden	524 * ± 79	1263 ± 69 *	39.21 ± 9.13 *	20.14 ± 5.38 *	33.41 ± 6.59 *
Immature	Control	505 ± 42 ^†^	1312 ± 180	17.56 ± 3.49 ^†^	4.23 ± 1.32 ^†^	14.05 ± 4.17 ^†^
	Iso/Aden	296 ± 43 *^,†^	512 ± 38 (39%) *^,†^	19.44 ± 8.59 ^†^	−12.00 ± 1.17 *^,†^	8.48 ± 1.86 ^†^

* significant change (*p* < 0.05) within selected age group, ^†^ *p* < 0.05 vs. the adult group.

## References

[1] Depre C., Taegtmeyer H. (2000). Metabolic aspects of programmed cell survival and cell death in the heart. Cardiovasc. Res..

[2] Eltzschig H.K., Eckle T. (2011). Ischemia and reperfusion—From mechanism to translation. Nat. Med..

[3] Khaliulin I., Clarke S.J., Lin H., Parker J.E., Suleiman M.S., Halestrap A.P. (2007). Temperature Preconditioning of Isolated Rat Hearts—A Potent Cardioprotective Mechanism Involving a Reduction in Oxidative Stress and Inhibition of the Mitochondrial Permeability Transition Pore. J. Physiol..

[4] Khaliulin I., Parker J.E., Halestrap A.P. (2010). Consecutive pharmacological activation of PKA and PKC mimics the potent cardioprotection of temperature preconditioning. Cardiovasc. Res..

[5] Bain J., Plater L., Elliott M., Shpiro N., Hastie C.J., McLauchlan H., Klevernic I., Arthur J.S., Alessi D.R., Cohen P. (2007). The selectivity of protein kinase inhibitors: A further update. Biochem. J..

[6] Khaliulin I., Halestrap A.P., Bryant S.M., Dudley D.J., James A.F., Suleiman M.S. (2014). Clinically-relevant consecutive treatment with isoproterenol and adenosine protects the failing heart against ischaemia and reperfusion. J. Transl. Med..

[7] Khaliulin I., Halestrap A.P., Suleiman M.S. (2011). Temperature Preconditioning is optimal at 26 °C and Confers Additional Protection to Hypothermic Cardioplegic Ischemic Arrest. Exp. Biol. Med..

[8] Dudley D.J., Suleiman M.S., Bond M., James A.F., Khaliulin I. (2014). 10 Cell-Permeable Cyclic AMP as a Novel Cardioprotective Agent. Heart.

[9] Pastorino J., Hoek J. (2008). Regulation of hexokinase binding to VDAC. J. Bioenerg. Biomembr..

[10] Chiara F., Castellaro D., Marin O., Petronilli V., Brusilow W.S., Juhaszova M., Sollott S.J., Forte M., Bernardi P., Rasola A. (2008). Hexokinase II detachment from mitochondria triggers apoptosis through the permeability transition pore independent of voltage-dependent anion channels. PLoS ONE.

[11] Pasdois P., Parker J.E., Halestrap A.P. (2012). Extent of mitochondrial hexokinase II dissociation during ischemia correlates with mitochondrial cytochrome c release, reactive oxygen species production, and infarct size on reperfusion. J. Am. Heart Assoc..

[12] Ostadalova I., Ostadal B., Kolar F., Parratt J.R., Wilson S. (1998). Tolerance to ischaemia and ischaemic preconditioning in neonatal rat heart. J. Mol. Cell. Cardiol..

[13] Riva E., Hearse D.J. (1991). Calcium and cardioplegia in neonates: Dose-response and time-response studies in rats. Am. J. Physiol..

[14] Modi P., Suleiman M.S. (2004). Myocardial taurine, development and vulnerability to ischemia. Amino Acids.

[15] Ostadal B., Ostadalova I., Kolar F., Sedmera D. (2014). Developmental determinants of cardiac sensitivity to hypoxia. Can. J. Physiol. Pharmacol..

[16] Imura H., Caputo M., Parry A., Pawade A., Angelini G.D., Suleiman M.S. (2001). Age-dependent and hypoxia-related differences in myocardial protection during pediatric open heart surgery. Circulation.

[17] Modi P., Imura H., Angelini G.D., Pawade A., Parry A.J., Suleiman M.S., Caputo M. (2003). Pathology-related troponin I release and clinical outcome after pediatric open heart surgery. J. Card. Surg..

[18] Ost’adalova I., Babicky A. (2012). Periodization of the early postnatal development in the rat with particular attention to the weaning period. Physiol. Res..

[19] Tseng Y.T., Kopel R., Stabila J.P., McGonnigal B.G., Nguyen T.T., Gruppuso P.A., Padbury J.F. (2001). Beta-adrenergic receptors (betaAR) regulate cardiomyocyte proliferation during early postnatal life. FASEB J..

[20] Zeiders J.L., Seidler F.J., Iaccarino G., Koch W.J., Slotkin T.A. (1999). Ontogeny of cardiac beta-adrenoceptor desensitization mechanisms: Agonist treatment enhances receptor/G-protein transduction rather than eliciting uncoupling. J. Mol. Cell. Cardiol..

[21] Giannuzzi C.E., Seidler F.J., Slotkin T.A. (1995). Beta-adrenoceptor control of cardiac adenylyl cyclase during development: Agonist pretreatment in the neonate uniquely causes heterologous sensitization, not desensitization. Brain Res..

[22] Swartz D.R., Zhang D., Yancey K.W. (1999). Cross bridge-dependent activation of contraction in cardiac myofibrils at low pH. Am. J. Physiol..

[23] Cross H.R., Opie L.H., Radda G.K., Clarke K. (1996). Is a high glycogen content beneficial or detrimental to the ischemic rat heart? A controversy resolved. Circ. Res..

[24] Kolocassides K.G., Seymour A.M., Galinanes M., Hearse D.J. (1996). Paradoxical effect of ischemic preconditioning on ischemic contracture? NMR studies of energy metabolism and intracellular pH in the rat heart. J. Mol. Cell. Cardiol..

[25] Shelley H.J. (1961). Glycogen reserves and their changes at birth and in anoxia. Br. Med. Bull..

[26] Young H.H., Shimizu T., Nishioka K., Nakanishi T., Jarmakani J.M. (1983). Effect of hypoxia and reoxygenation on mitochondrial function in neonatal myocardium. Am. J. Physiol..

[27] Lochner A., Genade S., Tromp E., Podzuweit T., Moolman J.A. (1999). Ischemic Preconditioning and the {beta}-Adrenergic Signal Transduction Pathway. Circulation.

[28] Asimakis G.K., Inners-McBride K., Conti V.R., Yang C.J. (1994). Transient beta adrenergic stimulation can precondition the rat heart against postischaemic contractile dysfunction. Cardiovasc. Res..

[29] Halestrap A.P., Pasdois P. (2009). The role of the mitochondrial permeability transition pore in heart disease. Biochim. Biophys. Acta.

[30] Kalogeris T., Baines C.P., Krenz M., Korthuis R.J. (2012). Cell Biology of Ischemia/Reperfusion Injury. Int. Rev. Cell Mol. Biol..

[31] Khaliulin I., Bond M., James A.F., Dyar Z., Amini R., Johnson J.L., Suleiman M.S. (2017). Functional and cardioprotective effects of simultaneous and individual activation of protein kinase A and Epac. Br. J. Pharmacol..

[32] Bergmeyer H.U. (1983). Methods of Enzymatic Analysis.

[33] hRiva E., Hearse D.J. (1991). The Developing Myocardium.

